# Abstracts DGT

**DOI:** 10.1515/iss-2019-2008

**Published:** 2019-03-20

## DGAV: MIS / Robotics

### Robotic thymectomy indicated for elderly patients with myasthenia gravis favors being free of concomitant disease and MGFA classification I

(Abstract ID: 655)

J. Rückert^1^

^1^*Charité - Universitätsmedizin Berlin - CCM*

**Background:**

Although complete thymectomy has become an indispensable treatment for patients with myasthenia gravis (MG), controversy still lies in the effectiveness of thymectomy in the elderly population. We aimed to identify predictive factors of the clinical outcome in elderly patients with MG undergoing thymectomy.

**Materials and methods:**

We performed a retrospective review on patients who underwent robotic thymectomy between 01/2003 and 12/2017, with at least one-year follow-up. Clinical outcomes were reported according to the Myasthenia Gravis Foundation of America Post-Intervention Status (MGFA-PIS). A "good outcome" was defined by complete stable remission (CSR), pharmacologic remission (PR) and minimal manifestations 0 (MM 0), and a "poor outcome" by minimal manifestations 1-3 (MM 1-3). Binary logistic regression models were used to determine the association of clinical parameters with "good outcome".

**Results:**

Sixty-eight (25 females, 43 males) of 580 patients who underwent robotic thymectomy for MG between 01/2003 and 12/2017 were older than 60 years at MG onset (median age at MG onset 67 years [range 61-85]). Four patients had less than 1-year of follow-up and 13 patients were lost to follow-up, leaving 51 patients for the final analysis with a median follow-up time of 60 months (range 12-263). The CSR rate was 7.8%, the improvement rate was 68.6% and the overall mortality rate was 11.8%. After excluding two patients seronegative for the anti-acetylcholine receptor (AChR) antibody, 10 out of 49 seropositive patients achieved "good outcome" (4 CSR, 3 PR and 3 MM-0). Achieving a good outcome was predicted by being free of concomitant disease (OR: 7.307, 95% CI (1.188-44.937), P=0.032) and MGFA classification I prethymectomy (OR: 6.696, 95% CI (1.259-35.620), P=0.026). Besides, the predictive factors remained significant when adding the two seronegative cases back to the series.

**Table:** 

Variable	Univariate OR (95% CI)	analysis P value	Multivariable OR (95% CI)	analysis P value
Age at onset, (yrs)	1.063(0.953-1.186)	0.27	NA	NA
Female	0.400(0.075-2.143)	0.285	NA	NA
BMI, (kg/m2)	0.891(0.725-1.095)	0.272	NA	NA
OAID free	4.500(0.513-39.436)	0.174	NA	NA
Being concomitant disease free a Purely ocular symptoms at onset	5.833(1.138-29.899)	0.034	7.307(1.188-44.937)	0.032
	5.575(1.076-30.720)	0.041	NA	NA
ThX delay (mons.),	0.958(0.877-1.046)	0.339	NA	NA
MGFA classification I	5.500(1.210-25.005)	0.027	6.696(1.259-35.620)	0.026
preThX Thymic follicular hyperplasia b Thymoma	1.333(0.113-15.704)	0.819	NA	NA
	1.095(0.262-4.572)	0.901	NA	NA

Binary logistic regression of clinical parameters associated with a good outcome in 49 thymectomized anti-AChR antibody seropositive MG patients

**Conclusion:**

Being free of concomitant disease and MGFA classification I prethymectomy appear to be predictors of "good outcome" after thymectomy in elderly MG patients.

## DGT/DGAV: Tracheo-oesophageal fistuals

### Management of intrathoracic esophageal fistula: a review of a thoracic high volume center

(Abstract ID: 433)

T. Plönes^1^, B. Hegedüs^1^, A. Slama^1^, K. Darwiche^1^, R. Karp-Wissel^1^, C. Taube^1^, C. Aigner^1^

^1^*Ruhrlandklinik Essen*

**Background:**

Intrathoracic esophageal fistula is a severe condition that can occur spontaneously, iatrogenic or secondary due to malignancy. In rare cases congenital fistulas remain asymptomatic until adulthood. Surgical repair or endoscopic management is complex and carries significant morbidity and mortality. We aimed to identify risk factors for failure of surgical repair.

**Materials and methods:**

We conducted a retrospective review of all patients with esophageal fistula diagnosed and treated in our department between 2006 and 2018. All data were extracted from the medical database of our department and further analysed.

**Results:**

We identified 26 patients (16 male, 10 female) with intrathoracic esophageal fistula during the observation period. In eight cases the underlying cause of the fistula was spontaneous/idiopathic or benign and in 18 cases associated to malignancy. In 14 cases the fistula was iatrogenic. A subgroup of 14 patients presented with a trachea-esophageal fistula, 10 patients with a drainage of the fistula in the pleural cavity and 2 in the mediastinum. In 22 cases direct closure of the defect via suture of the esophagus was performed. In 14 cases the closure of the fistula was performed in combination with a tracheal resection or suture of the membranous portion. In two cases esophageal diversion was the therapy of choice. In two cases the esophageal suture was covered by a diaphragmatic flap, in 14 cases by muscle (n= 6 M. latissimus dorsi , n=3 intercostal muscle, n=5 cervical muscle). In six cases complications occurred (postoperative bleeding, renal failure, chronic empyema, secondary wound healing /seroma, prolonged weaning). The median length of stay was 22 days (range between 3 and 105 days). The 30-day mortality was 7% (two of 26 patient died of sepsis).

**Conclusion:**

The surgical repair and endoscopic management is challenging in esophageal fistulas, but can be performed successfully. The treatment of this complex entity should be only performed in experienced centers with surgical and endoscopic expertise in all necessary techniques.

## DGT: ECMO - Extracorporeal membrane oxygenation

### Retrospective analysis of the Respiratory ECMO Survival Prediction (RESP) Score in 25 ARDS patients

(Abstract ID: 90)

S. Reindl^1^, A. Deljevic^1^, M. Beyer^1^, S. Raab^1^

^1^*Universitätsklinikum Augsburg*

**Background:**

The severe acute respiratory distress syndrome (ARDS) is characterized by a high mortality. For insufficient conventional ventilation extracorporeal membrane oxygenation (ECMO) is a well established, yet invasive and resource-intensive, therapeutical option nowadays. The Respiratory ECMO Survival Predicition (RESP) Score offers a clinical assessment to the outcome of the critically ill patients.

**Materials and methods:**

From 01/2015 to 09/2018 a total of 25 ECMO-systems (Maquet, Sweden) were established in patients suffering from ARDS of different origins. For 17 patients a veno-venous approach (outflow cannula V. femoralis, inflow cannula V. jugularis) was used, while in 8 patients a veno-arterial system (outflow cannula V. femoralis, inflow cannula A. femoralis) was set. The twelve defined parameteres by Schmidt et. al., 2014, prior to ECMO implantation were retrospectivly examined and the RESP Score (http://www.respscore.com) calculated. Actual survival was then compared to predicted survival based on the score results.

**Results:**

The most common cause for ARDS were bilateral pneumonias (n = 10) and fulminant or central pulmonary embolisms (n = 7). Preoperative PaO2/FiO2-quotient was 65 ± 16 in average, PaCO2 71 ± 27 mmHg respectivly. After an operational period of mean 7,9 ± 6 days (max. 22 days) a total of 13 patients could be weaned from ECMO therapy, while 12 patients died. All patients (average age 44,4 years, m:f = 16:9) were grouped in risk classes I to V based on the online calculated RESP score: I: 2, II: 3, III: 12, IV: 3, V: 5. Two patients survived in risk class I (actual survival 100% vs. predicted survival 92%), one patient in class II (33% vs. 76%), five in class III (42% vs. 57%), three in class IV (100% vs. 33%) and two in V (40% vs. 18%). Highest lethality was observed in patients suffering from a bilateral pneumonia (60%).

**Table:** 

risk class	I	II	III	IV	V
dead	0	2	7	0	3
weaned	2	1	5	3	2
% predicted	92%	76%	57%	33%	18%
% survival	100%	33%	42%	100%	40%

actual vs. predicted survival in risk classes I to V

**Conclusion:**

The RESP score provides reliable data on the assessment of outcomes and thus may be useful for indications on establishing ECMO therapy in ARDS patients. However, for the analyzed cohort even in the highest risk group patients benefit from the procedure by terms of survival. The RESP score can not fully reflect the patient’s pre-existing diseases and relevant clinical circumstances that may contribute to indivual survival. Established guidelines on patient selection and strategies on ECMO-implantation for failing conservative therapies of ARDS are still missing at the moment.

## DGT: Lung metastases

### Pulmonal Tularaemia mimicking Non Small Cell Lung Cancer

(Abstract ID: 38)

S. Taha-Mehlitz^1^, E.-M. Heising^1^, R. Schläpfer^1^, A. Leiser^1^

^1^*Luzerner Kantonsspital, Luzern*

**Background:**

Tularaemia is a bacterial zoonosis of the northern hemisphere. The infection is again required to be reported since 2004. In Switzerland more and more cases occurred during the last years (131 cases in 2017; 1.55/100.000). Francisella is a gram negative aerobic rod, especially the subspecies tularensis and holarctica occur in Central Europe. A seasonal accumulation during summer month is described. The infection is usually caused by animal contact (hares, rodents), ingestion of raw or half-cooked meat, contaminated water or inhalation of dust but also via vectors such as ticks and mosquitoes. The bacteria are highly contagious, 10-50 bacteria as an aerosol or intracutaneous application, or oral intake of about 100 may lead to an infection. A transmission between humans has never been observed. The incubation period is 3-5 (-21) days, mortality is 1%. Compared to 2016 the number of reported cases has doubled. The most common registered way of transmission is via ticks. Symptoms are fever, progressive infection of the entry site and lymphadenopathy. It is possible that a relevant proportion of infections may not be detected because of variable clinical appearance. Serological markers can be detected after one week at the earliest and last for years, so does immunity. Microbiological cultivation or polymerase chain reaction (PCR) is possible out of lymph nodes or bronchoalveolar lavage.

**Materials and methods:**

We report a case of a 78-year-old man, past professional racing cyclist, 30 pack-year smoking history. He takes low-dose cortisone for rheumatoid arthritis. As an incidental finding a tumor measuring 3.4 cm in the right middle lobe was found. It was fluorodeoxyglucose avid by PET scan with hiliar an mediastinal suspicious lymph nodes. The workup revealed no further tumor masses or metastasis. There was neither serological evidence nor hints in the sputum for tuberculosis. Due to suspicion of a malignant process middle lobe resection and lymphadenectomy were performed.

**Results:**

The frozen section showed a granulomatous inflammation. The PCR revealed F. tularensis. Ciprofloxacin was administered for two weeks. The postoperative course was uneventful. Early antibiotic therapy is crucial for the course of tularaemia. Fluoroquinolones, aminoglycosides, chloramphenicol, tetracyclines (second choice, high relapse rate) and rifampicin are effective. In Germany and Switzerland, macrolide-resistant strains are known, therefore an antibiogram should be done as soon as possible. Ciprofloxacin can be administered orally in mild forms of the infection. Severe progressions are treated with aminoglycosides and fluoroquinolones intravenously. The duration of therapy is 10-21 days. Ciprofloxacin or doxycycline can be used as postexposure prophylaxis for 7-14 days. No approved vaccine is available.

**Table:** 

form of infection	way of transmission	symptoms
ulceroglandular/glandular	contact to the bacteria even	lympadenopathy with or without
	without pre-existing lesion, bites of ticks/mosquitoes	preceding primary ulceration, abscessing or necrotizing lymph nodes
oculoglandulär	wiping with contaminated hand	one-sided conjunctivitis with
		edema of the lid or watery eye, photosensitivity and regional lymphadenopathy
oropharyngeal	ingestion of contaminated water	usually one-sided cervical or
	or food	submandibular lymphadenopathy, stomatitis, pharyngitis, tonsillitis, stomach pain, vomiting, diarrhea
pulmonal	inhalation of bacteria	bronchopneumonia, cough,
		chest pain, dyspnea, nausea, vomiting, hiliar lymphadenopathy

tularaemia: form of infection and according symptoms

**Conclusion:**

Our case showed an asymptomatic patient with pulmonary disease. In patients with local inflammation, ulcer formation, lymphadenopathy, indistinct fever and corresponding travel history or profession, tularaemia should be considered. The prognosis is generally favourable. Early diagnosis and antibiotic therapy are crucial for the course of the disease. Abscessed lymph nodes must be treated surgically.

**Picture:**
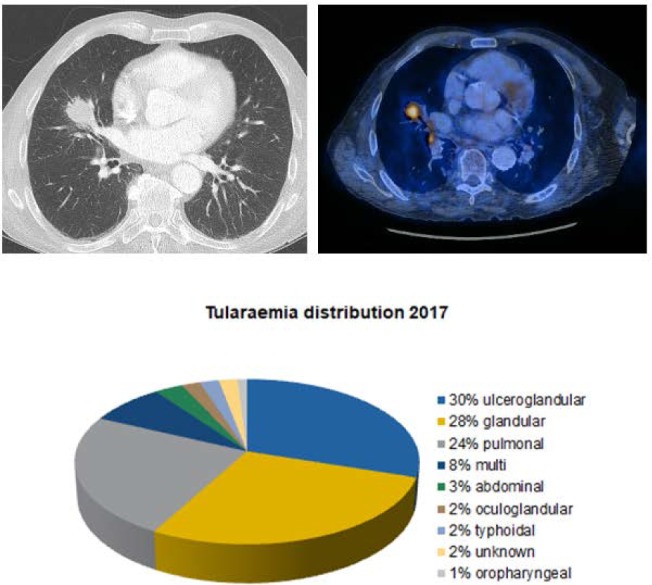
CT scan an PET scan showing a mass 3.4 cm in diameter in the right middle lobe and avide lymph nodes

### VATS (Video-assisted thoracoscopic surgery) lower lobe bisegmentectomy (S7/8) for a central pulmonary metastasis

(Abstract ID: 476)

A. Hiebinger^1^, F. Eicher^1^, T. Weik^1^, H. Mertins^1^, G. Gross^1^, J. Bodner^1^

^1^*Klinikum Bogenhausen, München*

**Background:**

Surgery for pulmonary metastasis is performed heterogeneously with regard to surgical approach (open versus VATS) and resection techniques (e.g. laser enucleation, electro-cautery resection, stapling). Complete tumor resection and preservation of lung parenchyma are of upmost importance. This is technically challenging, especially for central lesions close to vascular and bronchial segmental structures. Thus, simple thoracoscopic wedge resections are often not feasible.

**Materials and methods:**

Videopresentation of a thoracoscopic Bisegmentectomy (S7/8) of the left lower lobe for a central pulmonary metastasis

**Results:**

A VATS lower lobe bisegmentectomy (S7/8) was performed on a 62-year-old patient with a suspicious pulmonary nodules and a history of a deep anterior resection for a colorectal carcinoma. Different VATS techniques of vessel dissection and parenchymal control were applied. The postoperative course was uneventful. The patient was discharged on the 3rd postoperative day.

**Conclusion:**

VATS anatomic segmental resections represent a helpful tool in surgical therapy of central pulmonary metastasis.

### Cytoreductive surgery combined by intrathoracic hyperthermic chemoperfusion (HITHOC): a single center experience in mesothelioma patients

(Abstract ID: 692)

C. Grünewald^1^, L. Klotz^1^, E. Bulut^1^, F. Eichhorn^1^, F. Lasitschka^1^, H. Hoffmann^2^, H. Winter^1^, M. Eichhorn^1^

^1^*Thoraxklinik am Universitätsklinikum Heidelberg*

^2^*Klinikum Rechts der Isar der TU München*

**Background:**

The best surgical treatment for malignant pleural mesothelioma is still under debate. Lung sparing cytoreductive surgery by extended pleurectomy and decortication combined by HITHOC (CS-HITHOC) represents a promising treatment strategy that may be associated with reduced morbidity and improved survival. Aim of the study was to investigate feasibility and perioperative safety of the procedure conducted in a high volume center and to analyze overall survival.

**Materials and methods:**

A standardized CS-HITHOC procedure was established in 2014. Clinical data of all patients treated by CS-HITHOC were included in this retrospective analysis. Extended pleurectomy and decortication comprised total parietal and visceral pleurectomy. In case of transmural tumor infiltration diaphragm and pericardium were additionally resected and replaced using alloplastic materials. HITHOC was performed using a Rand Performer HT perfusion pump with 200mg cisplatin in isotonic saline. Intrathoracic target temperature was set to 42oC. Perfusion time was 60 min and flow was set to 1 l/min. Survival data were analyzed according to Kaplan-Meier. Data are given as mean±SD.

**Results:**

Between 2014 and 2018, 61 patients with pleural mesothelioma were treated by CS-HITHOC among 53 (87%) were male and 8 (13%) female. Mean age was 67±8 years (range 45-80 years). Predominant diagnosis was pleural mesothelioma with epitheloid subtype in 57 patients (93%), while four patients (7%) showed a biphasic tumor subtype. Perioperative, 30 and 60 day mortality was 0%, 90 day mortality was 1.6% related to rapid tumor progression. One-year survival rate in patients with a follow-up time of at least 18 months was 96%. Median overall survival was 42.2 months.

**Conclusion:**

Cytoreductive surgery in combination with HITHOC appears feasible and safe in mesothelioma patients with low perioperative morbidity and mortality. Initial survival data are promising, but for a more objective assessment a longer follow-up period has to be awaited.

## DGT: Pneumothorax

### Spontaneous ventilation leads to a significant reduction of procedure time in VATS bullectomy and pleurectomy

(Abstract ID: 819)

I. Metelmann^1^, J. Broschewitz^1^, S. Krämer^1^

^1^*Universitätsklinikum Leipzig*

**Background:**

Feasibility and safety of spontaneous ventilation (SV) video-assisted thoracoscopic surgery (VATS) bullectomy and pleurectomy in primary spontaneous pneumothorax (PSP) were assessed through a retrospective study.

**Materials and methods:**

This retrospective analysis involved 11 patients with spontaneous pneumothorax that needed a video-assisted thoracoscopic bullectomy and pleurectomy under general anesthesia with double-lumen endotracheal intubation (n=6) or spontaneous ventilation with laryngeal mask (n=5) between March 2017 and March 2018 at Leipzig University Hospital. Perioperative data of the two groups was compared.

**Results:**

The two groups matched concerning demographic profiles and medical condition. No conversions of anesthetic or surgical procedures were needed. No mortality occurred. Surgical time was not significantly different in both groups. However, induction time was significantly 16 minutes shorter when used spontaneous ventilation (p=0,001). No significant difference was seen in recovery time. Chest tube duration and time to discharge were comparable in both groups.

**Conclusion:**

Postoperative results, safety and recovery were comparable in SV-VATS and general anesthesia, while induction time was significantly shorter. SV-VATS seems a valid alternative in selected patients with PSP.

## DGT: Thymectomy

### Subxiphoid VATS Thymectomy

(Abstract ID: 623)

M. Hartert^1^, J. Tripsky^1^, M. Hürtgen^1^

^1^*Katholisches Klinikum Koblenz-Montabaur, Koblenz*

**Background:**

When endoscopic surgery is indicated for patients with myasthenia gravis and/or suspicion of thymomas or thymic carcinomas, most institutions use a lateral thoracic approach that - in an increasingly number of cases - includes robot-assisted surgery. However sophisticated it may be, with the unilateral thoracic approach, it can be difficult both to ensure the operative field in the neck and to identify the location of the contralateral phrenic nerve. As an alternative to the unilateral lack of visibility we hereby present the technique of minimally invasive thymectomy performed through the subxiphoid video-assisted thoracoscopic surgery (VATS) approach.

**Materials and methods:**

The patients were positioned supine on the operating table with a hyperextension of the patient’s neck. The whole dissection was performed through a 4-7cm transverse or longitudinal subxiphoid incision. In addition, two single 10mm thoracoscopic ports were inserted intercostally to the right and left chest cavities for videothoracoscope and subsequently for chest tubes. The sternum was elevated with two hooks connected to the sternal frame (Rochard bar, Aesculap). First, the lower hook was inserted through the subxiphoid incision. Following dissection of the mediastinal tissue including the major mediastinal vessels from the inner surface of the sternum, the superior hook was inserted thereafter percutaneously. With double elevation of the sternum the fatty tissue of the anterior mediastinum and the aortopulmonary window could be completely removed.

**Results:**

Starting in mid-July 2016, the study group includes 15 patients (six women and nine men) with a mean age of 66.1 years (range, 30-85 years). Myasthenia gravis was present in six patients (preoperative Osserman score was I-IIa). The mean operative time for thymectomy performed was 213 minutes (range, 121-303 minutes). The resected specimens have had a mean weight of 146 g (range, 27-300 g). Histological findings were as follows: thymic cyst in five patients, thymoma type AB (Masaoka-Koga I-IIb) in four patients, thymic hyperplasia in two patients and single patients with thymoma type A (Masaoka-Koga IIa), thymoma type B3 (Masaoka-Koga IIa), thymic carcinoma (Masaoka-Koga IIa) and parathyroid adenoma, respectively. Despite one patient, who suffered left side phrenic nerve lesion, there was neither postoperative morbidity nor mortality. Mean postoperative stay was 6.1 days (range, four to eleven days). Follow-up was uneventful by now with seven out of the 15 patients being followed up for more than twelve months.

**Conclusion:**

We conclude that the subxiphoid approach combined with bilateral single port intercostal VATS and double elevation of the sternum is a safe operative technique that enables very extensive thymectomy. Due to the insertion of the camera through a subxiphoid incision, the surgeons view is with the midline of the body and the surgical field is comparable to that of a median sternotomy. This makes it much easier to identify the location of the bilateral phrenic nerves and offer a good visualization in the neck area. In combination with low incidence of morbidity, short in-hospital stay and unremarkable follow-up we highly recommend this operative approach in the surgeons attempt to maximize minimal invasive techniques.

### VATS thymectomy versus sternotomy thymectomy in patients with thymoma – a retrospective single center study at a certified Myasthenia Center

(Abstract ID: 632)

A. Gassa^1^, S. Nienaber^1^, F. Dörr^1^, J. Seo^1^, M. Heldwein^1^, M. Schroeter^1^, K. Hekmat^1^, T. Wahlers^1^

^1^*Uniklinik Köln*

**Background:**

The best results in the therapy of thymus tumors can be seen in the surgical removal of the gland together with abnormal surrounding lymph nodes. Thymomas occur in almost half of the cases together with an autoimmune disease called myasthenia gravis. Patients who are associated with a pathological change in thymus tissue and myasthenia can benefit from surgical removal of the thymus. So far, thymomas have been removed by sternotomy. More minimally invasive methods show promising results. The objective of this study was to assess if the outcome of the video-assisted thoracoscopic surgery (VATS) approach was similar to open surgery approach at our Center for Myasthenia.

**Materials and methods:**

We retrospectively analyzed 86 consecutive patients with either resectable thymoma or thymic carcinoma at the Department of Cardiothoracic Surgery between 2009 and May 2018 who underwent thymectomy with the VATS or open approach. The patients were followed through physical examinations. Baseline characteristics were registered and hospital stay was analyzed as primary end point. Postoperative recurrence rate between the VATS or open approach was compared in patients with myasthenia.

**Results:**

Among 86 patients, 55 patients underwent VATS-thymectomy (64%) and 31 patients underwent thymectomy via sternotomy or hemisternotomy (36%). Patients’ mean age in the group of either VATS-thymectomy or sternotomy were 48 and 53 years. Body-Mass Index did not differ significantly in both groups (VATS-thymectomy: 25.97 ± 0.58 versus sternotomy: 26.17 ± 0.92). 38 of 55 patients undergoing VATS-thymectomy (69%) and 17 of 31 patients undergoing sternotomy (55%) had myasthenia gravis. Hospital stay differed significantly in both groups: patients after thymectomy via sternotomy stayed longer in hospital than those after VATS-thymectomy (10.61 ± 1.1 days versus 6.78 ± 0.5 days, p = 0.0008). Myasthenia relapse was observed in 2 of 38 patients after VATS-thymectomy (5%) and in 6 of 17 patients after thymectomy via sternotomy (35%).

**Conclusion:**

In our experience, minimal invasive thymectomy is applicable and feasible in patients with or without myasthenia gravis and thymoma.

### Left-sided extended robotic thymectomy in obese patients with myasthenia gravis

(Abstract ID: 647)

J. Rückert^1^

^1^*Charité - Universitätsmedizin Berlin - CCM*

**Background:**

Performing thoracic surgery in obese patients is complicated due to the obesity-related comorbidities and special body habitus. In addition, increased body mass could limit both the visualization and the motion range of the instruments. Previous studies have shown that obese patients with myasthenia gravis (MG) are more likely to develop postoperative complications, including myasthenic crisis. Da Vinci system creates a perfect performance in confined space due to the enhanced visualization, dexterity and precision. We aimed to assess the safety and feasibility of robotic thymectomy in obese patients with MG.

**Materials and methods:**

We performed a retrospective review of patients with MG who underwent robotic thymectomy from 01/2003-12/2017, with a body mass index (BMI) >= 30 kg/m2. The perioperative data were collected from the hospital information system and postoperative morbidity and mortality were reported according to Thoracic Morbidity and Mortality (TM&M) classification system.

**Results:**

Sixty-nine (BMI>= 30 kg/m2) of 580 patients who underwent robotic thymectomy for MG between 01/2003 and 12/2017 were eligible for inclusion. The weight of the specimens was 130 ± 75 g. In all cases, en-bloc resection without conversion was performed. Postoperative complications occurred in six (8.7%) patients. No postoperative mortality (grade V) was observed in our series. Grade II (requires pharmacologic intervention only) complications comprised 66.7% of all complications. One (16.7%) major complication (grade IIIa) that required the placement of a chest tube was due to postoperative chylothorax. Postoperative respiratory failure, leading to ventilation (grade IVa), was noted in a patient with a BMI of 51.1 kg/m2. In addition, postoperative MG crisis occurred in one patient (1.4%) who was treated by plasmapheresis.

**Table:** 

Gender	Age at onset, yrs	Preoperative BMI, kg/m2	Thymic pathology	Postoperative complications	Complication grade	Conconmitant disease
Male	55	32.3	Involution	Chylothorax	II	Hypertension
Male	46	31	Involution	Hypokalemia	II	NO
Male	57	31.1	Involution	Arrhythmia	II	CHD, DM, Hypertension, Arrhythmia
Male	66	30.1	Involution	Atrial fibrillation	II	CHD, DM, Hypertension, Arrhythmia
Male	72	34	Thymoma	Pleural effusion + MG crisis	IIIa	CHD, DM, Hypertension, Arrhythmia
Female	62	51.1	Thymoma	Respiratory failure	IVa	DM, Hypertension

Complications resulting from robotic thymectomy in 88 obese patients with MG (BMI≥ 30 kg/m2)

**Conclusion:**

Though technically challenging, robotic thymectomy is safe and feasible for obese patients with MG. Extra attention should be paid to the preoperative assessment, especially the history and the examinations of cardiac and pulmonary comorbidities.

### Pulmonary artery ligation in lobectomies and segmental resections. Titanium ligation clips- are they on par with traditional vascular ligation?

(Abstract ID: 677)

A. J. Merres^1^, E. Stoelben^1^

^1^*Kliniken Köln GmbH*

**Background:**

The use of clips for anatomical dissection in surgery is currently not validated by clinical studies. The authorisation for medical use in humans is solely based on technical specifications. Clips are helpful in endoscopic surgery as they are easy to apply, as well as being timesaving and cheap. We documented our experience with vascular dissection in video assisted thoracoscopic anatomical lung resection.

**Materials and methods:**

We analysed all anatomic lung resection by VATS from 2012 to 2017 regarding the early results until discharge of the patients from the hospital. We used linear endoscopic staplers (endogiaTM 30 - 45 mm, vascular medium (Medtronic) or echelon flexTM 45 mm, GST 45D), hand sutures (Premilene 5-0 or 4-0, 0,1-0,2 mm, B.Braun, running suture or single stitch) or clips (Aesculap® DS Titanium Ligation Clips, medium-large and large, Aesculap) for vascular dissection of the pulmonary artery. The primary end point was defined as postoperative bleeding resulting in the necessity of postoperative re- exploration or use of blood transfusions.

**Results:**

We identified 450 patents after lobectomy and 298 after segmental resections where patient records were complete. Stapler, hand suture and clips were used in 370, 83 and 293 procedures respectively. There were 20 cases of bleeding reported, 7 (1.9%) where a stapler was used to ligate the artery, 4 (4.8%) in patients with a sutured artery and 9 (1.2%) after the artery was clipped. 9 Patients were treated with blood transfusions, whereas 11 patients underwent re-exploration. There was no case of clip failure leading to bleeding. Overall there was no significant difference between the three groups regarding bleeding. There was also no difference between the three groups regarding complications in general. The procedure times for lobectomies and segmental resections were significantly shorter (140 minutes) with stapler- and clip use respectively in comparison to hand sutures (170 minutes) (p=0.0001). In segmental resection stapler use had a significantly shorter procedure time. There was no significant difference in thoracic drainage time for both lobectomies and segmental resections. The hospital stay for segmental resections was significantly shorter for patients with a clipped artery (7.5 days) in comparison to stapler use (9.2 days) (p=0.032), whereas there was no difference in lobectomies regarding hospital stay.

**Conclusion:**

Aesculap® DS Titanium Ligation Clips are a safe, fast and cheap way to ligate segmental pulmonary arteries compared to standard procedures like monofilament running suture or vascular stapling devices.

